# Draft genome sequence data of a tigecycline-resistant *Enterobacter cloacae* ST93 clinical strain isolated from bloodstream infection

**DOI:** 10.1016/j.dib.2018.10.004

**Published:** 2018-10-05

**Authors:** Long Sun, Juan Xu, Fang He

**Affiliations:** a Department of Clinical Laboratory, Hangzhou Hospital of Zhejiang Provincial Corps, Chinese People׳s Armed Police Forces, Hangzhou, Zhejiang 310051, China; b Institute of Hygiene, Zhejiang Academy of Medical Sciences, Hangzhou, Zhejiang 310013, China; c Department of Clinical Laboratory, Zhejiang Provincial People׳s Hospital, People׳s Hospital of Hangzhou Medical College, Hangzhou, Zhejiang 310014, China

**Keywords:** *Enterobacter cloacae*, Tigecycline-resistant, KPC-2 carbapenemase, Whole-genome sequencing

## Abstract

Here we report data on the draft genome sequence of a tigecycline-resistant *Enterobacter cloacae* ST93 clinical isolate TREC1 producing KPC-2 carbapenemase from China. The draft genome sequence of *E. cloacae* TREC1 consisted of 74 contigs that comprised 5,322,835 bp, and the overall GC content of this strain amounted to 54.63%. In total, 57 tRNA genes, 5 rRNA operons and 5108 protein-coding sequences were identified in the genome. TREC1 belongs to sequence type ST93. Nineteen antimicrobial resistance genes were confirmed. Antimicrobial susceptibility testing revealed that besides colistin this isolate is resistant to all antibiotics including tigecycline. This Whole Genome Shotgun project has been deposited at DDBJ/EMBL/GenBank under the accession number PJZE00000000. (http://www.ncbi.nlm.nih.gov/nuccore/PJZE00000000).

**Specifications table**

**Table:** 

Subject area	Biology
More specific subject area	Genomics, tigecycline-resistant *Enterobacter cloacae*
Type of data	Figure, table
How data was acquired	The data was acquired on HiSeq^TM^ 4000 (Illumina) sequencing platform
Data format	Analysed
Experimental factors	Genomic DNA from pure culture
Experimental features	Isolation of bacteria, genome sequencing, draft genome assembly and annotation
Data source location	Hangzhou, China
Data accessibility	Data is with this article, also this Whole Genome Shotgun project has been deposited at DDBJ/EMBL/GenBank under the accession number PJZE00000000.(http://www.ncbi.nlm.nih.gov/nuccore/PJZE00000000)

**Value of the data**

• This data may help us to understand the genomic feature and molecular characteristic of this bacterial pathogen.
• This data may help us to understand the resistance gene diversity of this bacterial pathogen.
• The genome sequence of *Enterobacter cloacae* TREC1 can be used as a reference sequence for comparative analysis of tigecycline-resistant *E. cloacae* aimed to reveal the mechanism of tigecycline-resistance in CRE.

## 1 Data

The draft genome sequence of *Enterobacter cloacae* TREC1 consisted of 74 contigs that comprised 5,322,835 bp, and the overall GC content of this strain amounted to 54.63%. In total, 57 tRNA genes, 5 rRNA operons and 5108 protein-coding sequences were identified by the RAST server, respectively. According to the MLST scheme of *E. cloacae*, TREC1 belongs to sequence type ST93. The genome also contains one intact and one incomplete prophage sequences, three CRISPR sequences and several IS elements: the majority belonging to the IS*3* and IS*5* families. The resistance genes present in the genome of the isolate are presented in Table 1. We identified the aminoglycoside resistance genes *armA*, *aadA5*, *strA* and *aph(6)-Id*; the beta-lactam resistance genes *bla*_CTX-M-14_, *bla*_ACT-7_, *bla*_KPC-2_, and *bla*_CTX-M-3_; the fluoroquinolone resistance genes *qnrS1* and *qnrA1*; the fosfomycin resistance gene *fosA*; the macrolide, Lincosamide and Streptogramin B resistance genes *msr(E)* and *mph(E)*; the phenicol resistance gene *catA2*; the sulphonamide resistance genes *sul1* and *sul2*; the trimethoprim resistance genes *dfrA1* and *dfrA14*; and the tetracycline resistance gene *tetA*. Ceftazidime, cefepime, imipenem, meropenem, piperacillin/tazobactam, cefoperazone/sulbactam, fosfomycin, amikacin, ciprofloxacin, trimethoprim/sulfamethoxazole, minocycline, tigecycline and colistin were used in the susceptibility testing. The isolate was resistant to all antimicrobials above except colistin. The MIC values are presented in Table 2. Isolate *E. cloacae* TREC1 not only produce KPC-2 carbapenemase, but are also resistant to tigecycline bringing a great challenge to clinical treatment. The relative expression level of efflux pump genes (*acrA*, *acrB*, *oqxA* and *oqxB*) in the tigecycline-resistant isolate TREC1 were examined by qRT-PCR. Relative expression of each target gene was calibrated against the corresponding expression of a tigecycline-susceptible isolate *E. cloacae* TSEC (expression = 1), which was served as the control. According to the results (Table 3), the expression level of efflux pump AcrAB was increased 2–3 fold relative to the susceptible isolate. The expression level of efflux pump OqxAB between TREC1 and TSEC was not significant. Tetracycline resistant gene *tetA* was found in TREC1, but no mutation was detected.

**Table 1:** Antimicrobial resistance genes in isolate *E. cloacae* TREC1.

**Resistance gene**	**%identity**	**HSP length/query**	**Contig**	**Position in contig**	**Predicted phenotype**	**Accession number**
Aminoglycoside						
*armA*	99.87	774/774	contig_57	1189.1962	Aminoglycoside resistance	AY220558
*aadA5*	100	789/789	contig_58	150.938	Aminoglycoside resistance	AF137361
*strA*	100	804/804	contig_59	5401.6204	Aminoglycoside resistance	M96392
*aph(6)-Id*	100	837/837	contig_59	6204.7040	Aminoglycoside resistance	M28829

Beta-lactam						
*bla*_CTX-M-14_	100	876/876	contig_85	1618.2493	Beta-lactam resistance	AF252622
*bla*_ACT-7_	99.56	1146/1146	contig_8	19,282.20427	Beta-lactam resistance	FJ237368
*bla*_KPC-2_	100	882/882	contig_54	3990.4871	Beta-lactam resistance	AY034847
*bla*_CTX-M-3_	100	876/876	contig_73	413.1288	Beta-lactam resistance	EF437434

Fluoroquinolone						
*qnrS1*	100	657/657	contig_7	23,019.23675	Quinolone resistance	AB187515
*qnrA1*	99.85	657/657	contig_70	54.710	Quinolone resistance	AY070235

Fosfomycin						
*fosA*	98.12	426/426	contig_27	322,842.323267	Fosfomycin resistance	AEXB01000013

MLS - Macrolide, Lincosamide and Streptogramin B						
*msr(E)*	100	1476/1476	contig_57	4261.5735	Macrolide, Lincosamide and Streptogramin B resistance	EU294228
*mph(E)*	100	885/885	contig_57	5792.6676	Macrolide resistance	EU294228

Phenicol						
*catA2*	96.11	642/642	contig_97	749.1390	Phenicol resistance	X53796

Sulphonamide						
*sul1*	100	840/840	contig_71	2179.3018	Sulphonamide resistance	AY224185
*sul2*	100	816/816	contig_36	3828.4643	Sulphonamide resistance	GQ421466

Tetracycline						
*tetA*	100	1200/1200	contig_83	1471.2670	Tetracycline resistance	AJ517790

Trimethoprim						
*dfrA1*	100	474/474	contig_58	1455.1928	Trimethoprim resistance	JQ690541
*dfrA14*	99.59	483/483	contig_7	30.512	Trimethoprim resistance	DQ388123

**Table 2:** MICs of the antibiotics tested in *E. cloacae* TREC1.

**Antibiotics**	**MIC(mg/L)**
ceftazidime^a^	256
cefepime^a^	>256
imipenem^a^	256
meropenem^a^	128
piperacillin/tazobactam^a^	>256
cefoperazone/sulbactam^a^	>256
fosfomycin^a^	>256
amikacin^a^	>256
ciprofloxacin^b^	>32
trimethoprim/sulfamethoxazole^b^	>32
minocycline^b^	16
tigecycline^c^	8
colistin^c^	0.25

a Tested by agar dilution method.

b Tested by Etest method.

c Tested by standard broth microdilution tests.

**Table 3:** Expression level of *acrA*, *acrB*, *oqxA* and *oqxB* in TREC1 compare with a tigecycline-susceptible isolate *E. cloacae* TSEC.

Isolate	Relative expression^a^				MIC (mg/L)^b^
	*acrA*	*acrB*	*oqxA*	*oqxB*	
TSEC	1	1	1	1	0.25
TREC1	2.86 ± 0.25	3.07 ± 0.29	1.24 ± 0.18	1.06 ± 0.21	8

a Relative expression compared with tigecycline-susceptible isolate TSEC (expression = 1). Results are means of 3 runs ± standard deviation.

b MIC of tigecycline.

## 2 Experimental design, materials and methods

**Fig. 1:**
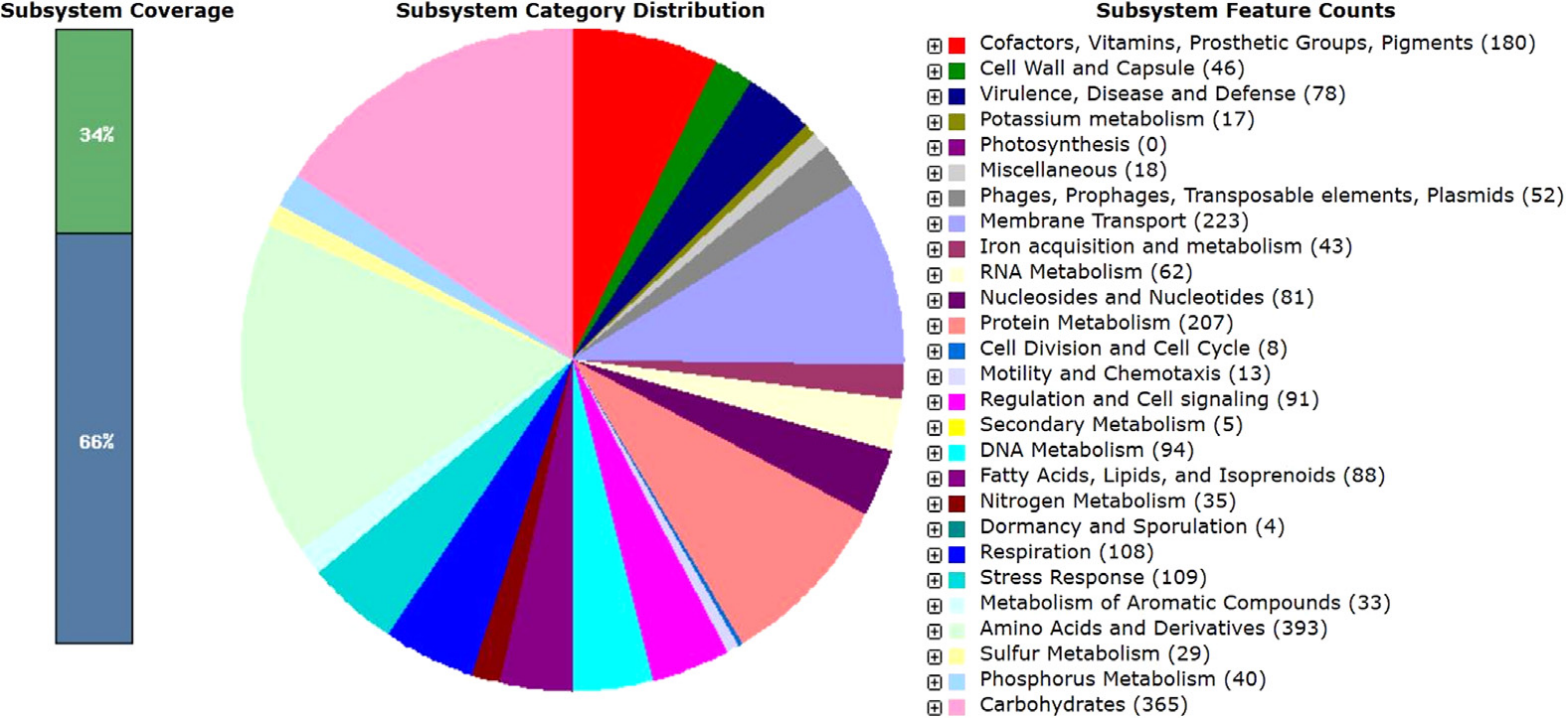
An overview of the subsystem categories assigned to the genome of *Enterobacter cloacae* TREC1. The whole genome sequence of the strain TREC1 was annotated using the Rapid Annotation System Technology (RAST) server. The pie chart demonstrates the count of each subsystem feature and the subsystem coverage.

Isolate *E. cloacae* TREC1 was recovered from a blood sample of a male hospitalised patient in Hangzhou, Zhejiang province, China, in 2017. The isolate was preliminarily identified using the VITEK 2 system (bioMérieux, France) and was further confirmed by 16S rRNA gene sequencing. Antimicrobial susceptibility testing was performed according to the guidelines of the Clinical and Laboratory Standards Institute (CLSI). The MICs of tigecycline and colistin were determined using standard broth microdilution tests with fresh (<12 h) Mueller-Hinton broth (Cation-adjusted, Oxoid LTD, Basingstoke, Hampshire, England). The MICs of other antimicrobial agents were determined using the agar dilution method and Etest method. Whole genome sequencing has increasingly being applied to clinical practice server [1]. The genome of *E. cloacae* TREC1 was sequenced using the Illumina HiSeq^TM^ 4000 platform (Illumina Inc., San Diego, CA, USA) following the paired-end 2 × 150 bp protocol. The whole genome sequence was assembled using CLC Genomics Workbench 10.0 software (Qiagen, Valencia, CA) and annotated by the Rapid Annotation System Technology (RAST) server [2]. The pie chart demonstrated the counts for each subsystem feature and the subsystem coverage (Fig. 1). *In silico* Multilocus sequence typing (MLST) analysis was performed using the PubMLST database. Resistance-related genes were analysed using ResFinder 3.0 [3]. Further bioinformatics analysis, such as identification of insertion elements (IS), prophage sequences and clustered regularly interspaced short palindromic repeat (CRISPR) sequences were predicted by application of ISfinder, PHASTER and CRISPRFinder, respectively [4], [5], [6].

## Funding

This study was supported by grants from the National Natural Science Foundation of China (81702042) and the Zhejiang Provincial Medical and Health Science and Technology plan (2018KY344).
