# Supplementary material for: Draft genome sequence data of a tigecycline-resistant Enterobacter cloacae ST93 clinical strain isolated from bloodstream infection
Source: Data Brief. 2018 Oct 5;21:414–8. doi: 10.1016/j.dib.2018.10.004 (PMC6198125; doi:10.1016/j.dib.2018.10.004)
Supplement: Supplementary file 1 — Supplementary material [file mmc1.docx]

**Conflict of interest: none**
